# Case Report: BAF-Opathies/SSRIDDs Due to a *de novo* ACTL6A Variant, Previously Considered to Be Heart-Hand Syndrome

**DOI:** 10.3389/fcvm.2021.708033

**Published:** 2021-08-13

**Authors:** Zhuang-Zhuang Yuan, Xiao-Hui Xie, Heng Gu, Wei-Zhi Zhang, Yi-Qiao Hu, Yi-Feng Yang, Zhi-Ping Tan

**Affiliations:** ^1^Department of Cardiovascular Surgery, Clinical Center for Gene Diagnosis and Therapy, The Second Xiangya Hospital of Central South University, Changsha, China; ^2^Department of Cell Biology, School of Life Sciences, Central South University, Changsha, China; ^3^Hunan Key Laboratory of Animal Models for Human Diseases, School of Life Sciences, Central South University, Changsha, China

**Keywords:** heart-hand syndromes, BAF-opathies, SSRIDDs, Actl6a, craniofacial dysmorphisms, intellectual disability

## Abstract

**Purpose:** This study aims to identify genetic lesions in patients with congenital heart disease (CHD) with or without other phenotypes. In this study, over 400 patients were recruited and several novel variants in known causative genes were identified. A Chinese patient clinically diagnosed with HHS (patent ductus arteriosus, persistent left superior vena cava, and congenital absence of left arm radius) was included in the study cohort.

**Methods:** Targeted, whole exome, and Sanger sequencing were performed to identify genetic lesions. The effects of the variant on ACTL6A RNA and protein were assessed using bioinformatics analysis.

**Results:** At the start of the study, no mutations in known and candidate causative genes associated with CHD were identified. Seven years later, we noticed craniofacial deformities and identified a *de novo* heterozygous deletion variant in ACTL6A (NM_004301, c.478_478delT; p.F160Lfs^*^9). Intellectual disability and short stature were identified by a follow-up visit 10 years later. This variant leads to frameshift sequences and a premature termination codon and may affect the features of proteins. According to the nonsense-mediated mRNA decay theory, this variant may induce the decay of ACTL6A mRNA in patients.

**Conclusion:** Our study reported the first ACTL6A variant in a Chinese individual, providing further evidence that ACTL6A is involved in heart and upper limb skeletal and intellectual development, thereby expanding the spectrum of ACTL6A variants. Thus, mutation analysis of the ACTL6A gene should be considered in patients with BAF-opathies or heart-hand syndromes due to potential misdiagnosis. Craniofacial dysmorphisms and intellectual disability are key to distinguishing these two diseases clinically, and attention to developmental delay/intellectual disability and craniofacial deformities will contribute to the diagnosis of BAF-opathies.

## Introduction

Heart-hand syndrome (HHS) is a class of autosomal dominant hereditary diseases characterized by upper limb skeletal deformities and congenital cardiac defects. According to the different combinations of heart and upper limb defects, heart-hand syndromes can be classified roughly into four categories (1).

The most common type is Holt-Oram syndrome (MIM# 142900), which is composed of skeletal radial ray abnormalities and cardiac malformations that mainly include atrial and/or ventricular septal defects. The skeletal defects may be unilateral and asymmetrical and vary from subtle to frank phocomelia. Incidence is estimated to be around 1 in 100,000 individuals (2). Deleterious mutations in TBX5 and SALL4 are the genetic lesions of Holt-Oram syndrome (3).

HHS II (Tabatznik's syndrome) is rare and characterized by upper limb malformations (hypoplastic deltoids; skeletal anomalies in the humeri, radius, ulnae, and thenar bones; and brachydactyly type D) and congenital cardiac arrhythmias (1).

HHS III (Spanish type) is poorly understood and characterized by cardiac conduction diseases and brachydactyly type C (4).

The potential HHS IV (Slovenian type) was first proposed in 2005 and characterized by arrhythmia, dilated cardiomyopathy (DCM), and a unique type of brachydactyly with mild hand and severe foot malformations (5).

BRG1-associated factor chromatin remodeling complex (BAF complex, or SWI/SNF) disorder is a specific group of developmental delay (DD)/intellectual disability (ID) syndromes with distinctive craniofacial dysmorphisms, variable congenital anomalies, and acral defects, also termed “BAF-opathies” and “SWI/SNF-related intellectual disability disorders” (SSRIDDs), such as Coffin-Siris syndrome (6, 7). Actin-like 6A (ACTL6A, MIM# 604958), members of BAF complex, have been reported as causative genes in four BAF-opathy individuals (8, 9).

## Materials and Methods

Whole peripheral blood samples from the patient and his family were obtained and stored in ethylenediaminetetraacetic acid tubes. Genomic DNA was extracted using the QIAamp DNA Blood Mini Kit (250) (Qiagen, Valencia, CA, USA).

The primers were designed using PrimerQuest and NCBI-Primer design tools. PCR conditions consisted of 94°C for 30 s, 55°C for 30 s, and 72°C for 1 min, for a total of 35 cycles using 2 × Power Taq PCR MasterMix (BioTeke, Beijing, China). PCR products were electrophoresed on a 1% agarose gel. The PCR fragment was subsequently cut, and the purified fragments were sequenced on a 3730XL sequencer (Applied Biosystems).

Targeted sequencing and whole-exome sequencing (WES) were mainly conducted by the BerryGenomics Bioinformatics Institute. The gene panel of targeted sequencing contained a series of known and candidate genes associated with congenital heart disease. Exomes were captured using Agilent SureSelect Human All Exon V6 kits and sequenced on an Illumina NovaSeq6000 (Illumina Inc., San Diego, USA). The WES data filtration strategy was based on previously published literature (10).

## Results

### Clinical Phenotypes

**Figure 1:**
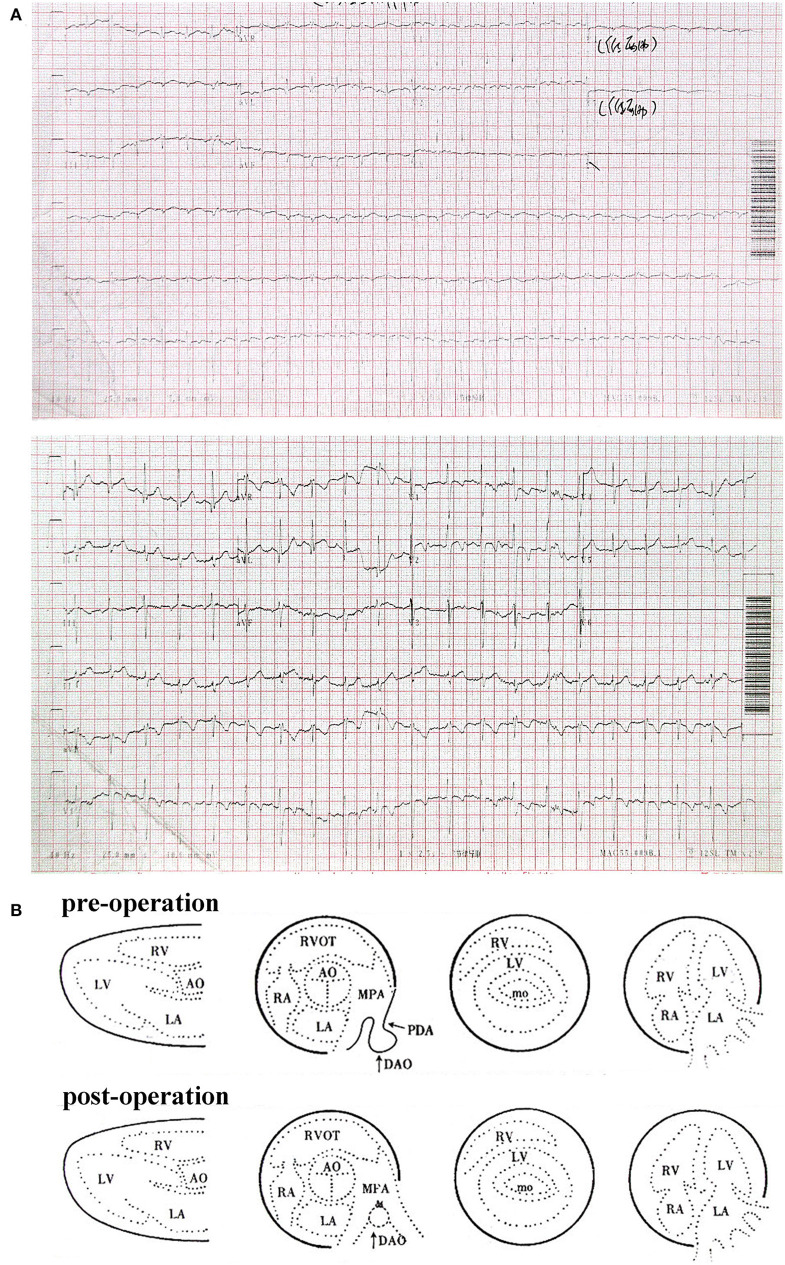
**(A)** Electrocardiogram of patient at 3 years old. **(B)** Color Doppler echocardiography report of patient pre- and post-operation.

**Figure 2:**
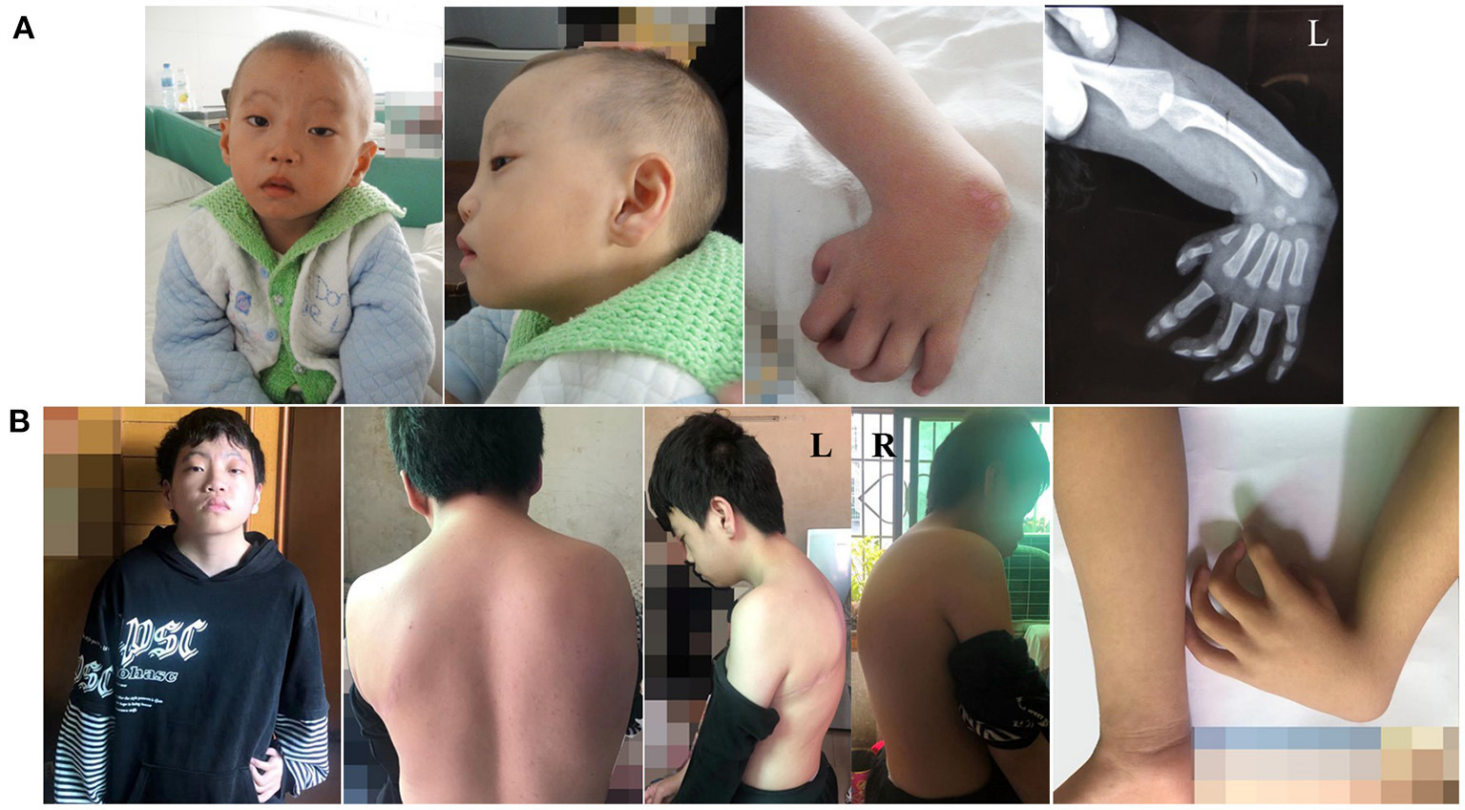
**(A)** Facial and left-hand appearance of affected individual at 3 years old. **(B)** Facial, left-hand, and dorsum appearance of affected individual at 14 years old.

**Figure 3:**
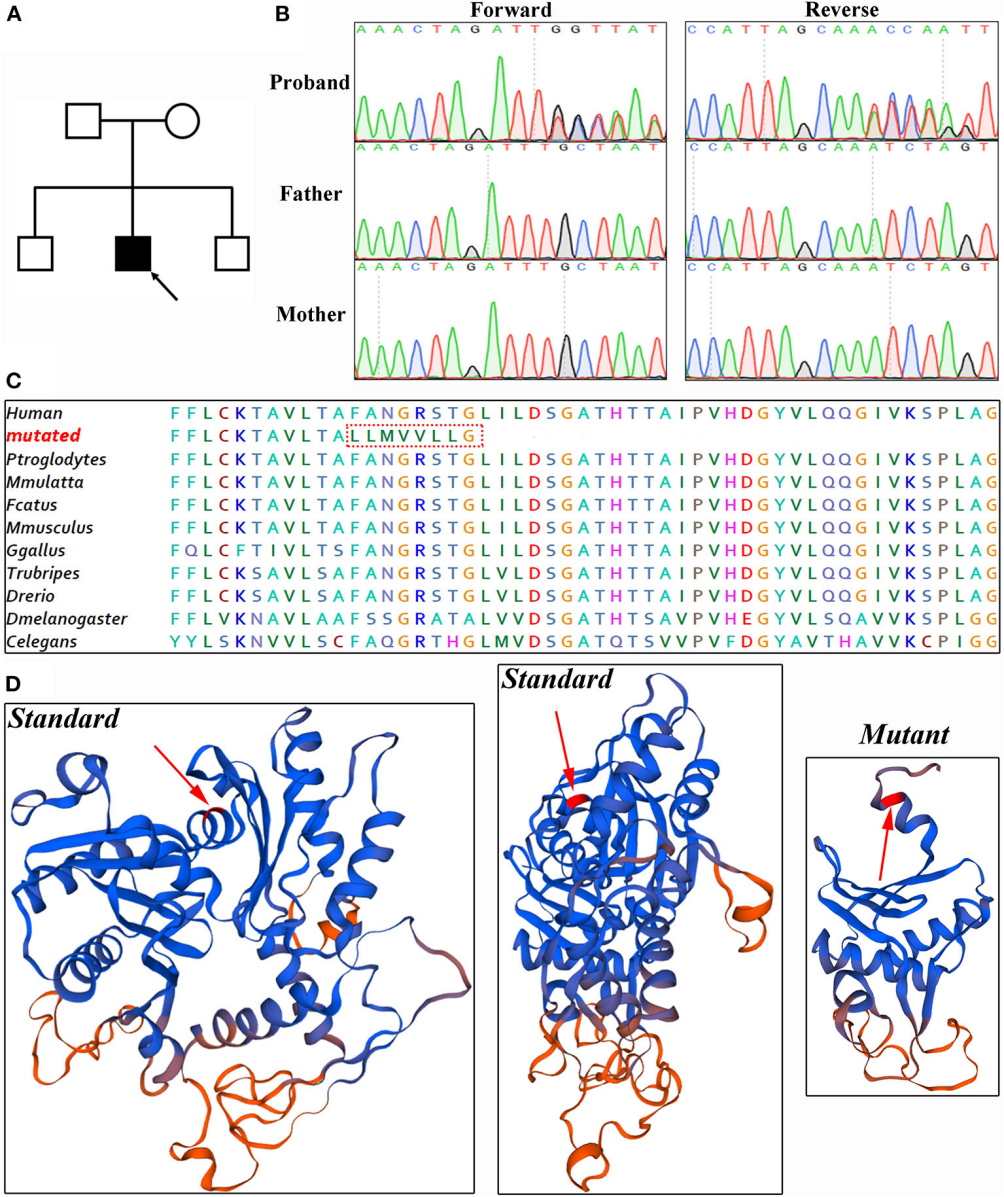
Genetic analysis. **(A)** Pedigree of patient. Solid black, affected. **(B)** Sanger sequencing verified this *de novo* variant. **(C)** Amino acid alignment analysis showed ACTL6A protein is evolutionarily highly conserved. **(D)** Referenced and mutated ACTL6A protein model.

The proband was born in 2007, weighing 2,600 g with 49 cm in length after a gestation period of 39 weeks. The patient was weak and susceptible to illness as a child. A heart murmur was detected in the patient at a local hospital at the age of 1. For further treatment, his family requested hospitalization when he was 3 years old. After examination, he was diagnosed with HHS (patent ductus arteriosus, persistent left superior vena cava, and congenital absence of left arm radius) (Figures 1A, 2A). PDA ligation under general anesthesia was performed in the patient, and the procedure was successful (Figure 1B). After obtaining consent from the patient's parents, we recruited their blood and inquired about their family and previous medical history (Figure 3A). In addition, craniofacial dysmorphisms were observed in the patient. Ten years later, we visited the patient and inquired about his physical and study conditions in detail. At the time of the visit, the patient was 152 cm tall, weighed 42 kg, and was able to communicate normally. His grades were between 20 and 40 in all subjects (total score 100–120/subject), near the bottom of his class. According to the latest version of “the height and weight percentile table of Chinese people aged 0–18 years” released by the Capital Pediatric Research Institute, the height and weight of the proband were lower than 90 and 80% of the same age group (13.5 years old), respectively. Mild intellectual disability (IQ 59) was detected using the Wechsler Intelligence Scale. An imbalance of the dorsal muscles was observed (Figure 2B). Combined with sequencing results, we concluded that the proband had BAF-opathies.

### Genetics Analysis

Given that the patient's clinical diagnosis was heart-hand syndrome, we screened the *TBX5, SALL4*, and *LMNA* genes, but no deleterious variation was found. Subsequently, targeted sequencing, including a panel of genes associated with congenital heart disease, was performed on the patient.

Whole-exome sequencing was conducted to generate 12.4 GB data with 99% coverage and a depth of >100 × in this patient. After data filtration, a novel heterozygous variant of ACTL6A (NM_004301, c.478_478delT; p.F160Lfs^*^9) was identified. This variant led to frameshift sequences and a premature termination codon, potentially affecting the features of the resulting proteins. According to the nonsense-mediated mRNA decay theory, this variant may induce the decay of ACTL6A mRNA in patients. Bioinformatics analysis predicted that this variant was deleterious.

Sanger sequencing was performed to validate this variant (Figure 3B). Cosegregation analysis showed that this is a *de novo* variant and does not exist in the 1,000 Genome Browser, ExAC Browser, Exome Variant Server, gnomAD browser, or 200 unrelated ethnically matched healthy controls. The amino acid sequence was highly conserved (Figure 3C). There is a large distinction between the standard and mutant protein models constructed with SWISS-MODEL, which may affect the assembly of the BAF complex (11) (Figure 3D).

## Discussion

Clinical diagnosis is crucial for etiology and genetic counseling. In this study, based on patent ductus arteriosus, persistent left superior vena cava and congenital absence of left arm radius, the proband was diagnosed with HHS, while craniofacial deformities and ID were ignored. Seven years later, combined with sequencing results, we initially diagnosed the patient with a new and recently reported BAF-opathy and confirmed ID and imbalance of dorsal muscles at a follow-up visit (8, 9).

To the best of our knowledge, two studies involving four patients with ACTL6A variants have been previously reported. The variants included p.Arg377Trp (two patients), p.Glu227Gln, and r.1134_1221del. DD/ID is a common phenotype in all cases; only two individuals with the p.Arg377Trp variant manifest heart defects and hernias (8). Craniofacial dysmorphisms are also common but may be mild and non-specific. In addition, other congenital defects, such as toes and finger syndactyly/clinodactyly/overriding, hypospadias, laryngomalacia, and renal alterations were observed (9).

Mutations in genes encoding components of the BAF complex, such as ARID1B, ARID1A, ARID2, SMARCA2, SMARCA4, SMARCB1, SMARCC2, SMARCE1, SOX11, DPF2, and BICRA, cause a range of disorders, including syndromic intellectual disability, Coffin-Siris syndrome (CSS), and Nicolaides-Baraitser syndrome (NCBRS) (6, 12–18). Two terms, “SWI/SNF related intellectual disability disorders” (SSRIDDs) and “BAF-opathies,” have been proposed to represent the full range of these diseases (8, 10).

In conclusion, we identified a novel deletion variant that caused a truncated sequence and may induce the decay of ACTL6A mRNA, according to the nonsense-mediated mRNA decay theory. The symptoms of our patient were highly consistent with BAF-opathies and had a more severe acral phenotype. As common phenotypes of BAF-opathies and heart-hand syndrome, congenital heart defect and acral deformity are more noticeable, making it easier to diagnose this syndrome. Thus, a focus on DD/ID and craniofacial deformities is likely to contribute to the diagnosis of BAF-opathies and SSRIDDs.

## Data Availability Statement

The datasets presented in this study can be found in online repositories. The names of the repository/repositories and accession number(s) can be found below: The Sequence Read Archive (SRA).

## Ethics Statement

The studies involving human participants were reviewed and approved by Ethics Committee of the Second Xiangya Hospital of the Central South University. Written informed consent to participate in this study was provided by the participants' legal guardian/next of kin. Written informed consent was obtained from the minor(s)' legal guardian/next of kin for the publication of any potentially identifiable images or data included in this article.

## Author Contributions

Z-PT: conceptualization. Z-PT and Z-ZY: data curation and formal analysis. Z-PT and Y-FY: funding acquisition. Z-ZY and X-HX: investigation. Z-ZY, HG, and Y-QH: validation and methodology. Z-ZY: writing—original draft. Z-PT, Z-ZY, X-HX, HG, W-ZZ, Y-QH, and Y-FY: writing—review and editing. All authors contributed to the article and approved the submitted version.

## Conflict of Interest

The authors declare that the research was conducted in the absence of any commercial or financial relationships that could be construed as a potential conflict of interest.

## Publisher's Note

All claims expressed in this article are solely those of the authors and do not necessarily represent those of their affiliated organizations, or those of the publisher, the editors and the reviewers. Any product that may be evaluated in this article, or claim that may be made by its manufacturer, is not guaranteed or endorsed by the publisher.
